# Symposiums on Practical Medical Issues

**Published:** 1916-04

**Authors:** 


### SYMPOSIUMS ON PRACTICAL MEDICAL ISSUES.

---

This method of collating professional opinion has been followed by many medical journals and to some extent by this one. Some weeks ago, Dr. Wm. H. Heath suggested that the regular establishment of a department of this nature would be of interest to our readers and we immediately ordered a thousand letter heads to be used in securing discussion of special topics by authorities. Obviously, success in such a movement depends largely on the combined favor and leisure of men upon whom we have no claim for the former and whose ability to speak with authority is not compatible with an abundance of the latter. Nevertheless, our experience covering many years is that professional men are generous with their accumulated knowledge and that the busy man finds time to do many things for which the man of comparative leisure has no time. As regularly as possible, we shall publish symposiums on questions of interest and we ask the co-operation of our readers in formulating questions and starting discussions. Here, for example, is a question recently raised by a doubting Thomas: Is there a definite haemophilia actually corresponding to the text book descriptions and conforming to the stated peculiarities of heredity? This sceptic does not deny that obstinate bleeding occurs and that it is difficult or impossible of control, but he questions whether it is strictly idiosyncratic, believing that it is due to more or less temporary pathologic conditions, and he states that he has never been able to trace the peculiar hereditary chain of transmission described as typic of haemophilia. Without expressing a personal opinion, we would welcome reports, for or against, and suggestions of authorities who might be asked for opinions.

### Quarantine of Infectious Cases.

The following questions were submitted to the Health Departments of a number of cities: "In what diseases is dismissal from quarantine based on negative bacteriologic evidence and in what on an arbitrary time limit? Is there any evidence that the latter method is not efficient or, on the other hand, that quarantine is unnecessarily prolonged? Is the former method applicable to typhoid? If not, what are the practical obstacles?" The letter head alluded to, expressly waived an insistence on categoric answers, as we felt that nothing so much obscures the truth as such a method—it being a matter of common observation that the questioning of witnesses in courts and the insistence on answers limited strictly to the questions as put often, if not usually, prevents the witness from fulfilling his oath to tell "the whole truth and nothing but the truth." The promptness and fullness of replies, and the number of replies received, in proportion to the number of letters sent, was most gratifying.

